# Supplementary material for: Exploring variables associated with medication non-adherence in patients with type 2 diabetes mellitus
Source: PLoS One. 2021 Aug 23;16(8):e0256666. doi: 10.1371/journal.pone.0256666 (PMC8382191; doi:10.1371/journal.pone.0256666)
Supplement: S1 Appendix — (DOCX) [file pone.0256666.s001.docx]

**Appendix A.1**

**Medication adherence questionnaire (Arabic)**

**مقياس موريسكي لتحديد درجة الالتزام بأخذ الدواء**

**يرجى الاجابة ب نعم او لا على الاسئلة التالية**

1. هل تنسى أخذ الدواء؟
2. هل تلتزم باوقات أخذ الدواء؟
3. هل تترك أخذ الدواء عندما تشعر بتحسن؟
4. هل تترك أخذ الدواء إذا شعرت بأن حالتك أصبحت أسوأ؟
