# Supplementary material for: Exploring variables associated with medication non-adherence in patients with type 2 diabetes mellitus
Source: PLoS One. 2021 Aug 23;16(8):e0256666. doi: 10.1371/journal.pone.0256666 (PMC8382191; doi:10.1371/journal.pone.0256666)
Supplement: S2 Appendix — (DOCX) [file pone.0256666.s002.docx]

**Appendix A.2**

**Medication adherence questionnaire (English)**

**Please answer the following questions with yes or no:**

**Ever forget to take medicines (Yes/No)**

**Ever careless about taking medicines (Yes/No)**

**Stop taking medicines when feeling better (Yes/No)**

**Stop taking medicines if you feel worse (Yes/No)**
