# Supplementary material for: Exploring variables associated with medication non-adherence in patients with type 2 diabetes mellitus
Source: PLoS One. 2021 Aug 23;16(8):e0256666. doi: 10.1371/journal.pone.0256666 (PMC8382191; doi:10.1371/journal.pone.0256666)
Supplement: S3 Appendix — (DOCX) [file pone.0256666.s003.docx]

**Appendix B.1**

**Beliefs about Medications (BMQ)-General questionnaire (Arabic)**

| **وجهات نظرك حول الأدوية الموصوفة لك** |
| --- |

- نود أن نسألك عن وجهات نظرك الشخصية حول الأدوية الموصوفة لك.
- يرجى اظهار كم كنت تتفق أو تختلف معهم وذلك بوضع علامة في الخانة المناسبة.

**لا توجد إجابات صحيحة أو خاطئة.
نحن مهتمون في وجهات النظر الشخصية الخاصة بك**

| **لا أوافق بشدة** | **لا أوافق** | **غير متأكد** | **أوافق** | **أوافق بشدة** | **وجهات النظر حول الأدوية الموصوفة لك:** |
| --- | --- | --- | --- | --- | --- |
|  |  |  |  |  | صحتي، في الوقت الحاضر، تعتمد على أدويتي* |
|  |  |  |  |  | *حقيقة الحاجة إلى تناول الأدوية تقلقني |
|  |  |  |  |  | *حياتي ستكون مستحيلة من دون أدويتي |
|  |  |  |  |  | *أنا أقلق أحيانا بشأن الآثار الطويلة الأجل لأدويتي |
|  |  |  |  |  | *بدون أدويتي سأكون مريضا جدا |
|  |  |  |  |  | *أدويتي تشكل لغزا بالنسبة لي |
|  |  |  |  |  | *صحتي في المستقبل سوف تعتمد على أدويتي |
|  |  |  |  |  | *أدويتي تعطل حياتي |
|  |  |  |  |  | *أحيانا أنا أقلق من أن أصبح معتمدا بشكل كبير على أدويتي |
|  |  |  |  |  | *أدويتي تحميني من أن أصبح أسوأ |
