# Supplementary material for: Exploring variables associated with medication non-adherence in patients with type 2 diabetes mellitus
Source: PLoS One. 2021 Aug 23;16(8):e0256666. doi: 10.1371/journal.pone.0256666 (PMC8382191; doi:10.1371/journal.pone.0256666)
Supplement: S4 Appendix — (DOCX) [file pone.0256666.s004.docx]

**Appendix B.2**

**Beliefs about Medications (BMQ)-General questionnaire (English)**

| **Statements** | **Totally**  **agree** | **Agree** | **Neutral** | **Disagree** | **Totally disagree** |
| --- | --- | --- | --- | --- | --- |
| My health, at present, depends on my medicines |  |  |  |  |  |
| My life would be impossible without my medicines |  |  |  |  |  |
| Without my medicines I would become very ill |  |  |  |  |  |
| My health in the future will depend on my medicines |  |  |  |  |  |
| My medicines protect me from becoming worse |  |  |  |  |  |
| Having to take medicines worries me |  |  |  |  |  |
| I sometimes worry about the long-term effects of my medicines |  |  |  |  |  |
| My medicines are a mystery to me |  |  |  |  |  |
| My medicines disrupt my life |  |  |  |  |  |
| I sometimes worry about becoming too dependent on my medicines |  |  |  |  |  |
