# Supplementary material for: Exploring variables associated with medication non-adherence in patients with type 2 diabetes mellitus
Source: PLoS One. 2021 Aug 23;16(8):e0256666. doi: 10.1371/journal.pone.0256666 (PMC8382191; doi:10.1371/journal.pone.0256666)
Supplement: Attachment — Submitted filename: Comments.docx [file pone.0256666.s005.docx]

**Comments to editor and authors**

Thank you for submitting this interesting manuscript that that evaluated the factors associated with non-adherence among diabetic Jordanians. The study is well written however, I have few comments that should be addressed.

-Major point:

Add sample size calculations.

-Minor point:

Please identify the study type in the methods (abstract and manuscript).

Please clarify that necessity and concerns are the BMQ parts in the method-abstract.

Please add OR values to the results section in the abstract

Line 120: The sentence is not clear.

The abbreviation of Hemoglobin A1c% is HbA1c%, please modify the manuscript accordingly.

In table 2 footnotes, please add the CCBs abbreviation clarification.

Line 26: add a coma after information.

Line 33: add “a” before significant predictor.

Line 199: Remove “a” before studies.

Line 204: remove “on”
